# Blockchain, Artificial Intelligence, and Cyber Defense on Sensor Networks

**DOI:** 10.3390/s26092762

**Published:** 2026-04-29

**Authors:** Hiroshi Watanabe

**Affiliations:** Department of Electrical and Computer Engineering (EE), National Yang Ming Chiao Tung University, No. 1001, Daxue Rd. East Dist., Hsinchu City 300093, Taiwan; hwhpnabe@nycu.edu.tw

**Keywords:** Internet of Things (IoT), Blockchain of chips, fake news, spoofing, physical artificial intelligence, cyber defense

## Abstract

**Highlights:**

Physical Cyber Authentication (PCA) is akin to a physically unclonable function (PUF) without using a dedicated System-on-a-Chip (SoC). PCA is free from disadvantages attributable to such SoCs, like temperature dependence, limited lifetime and endurance, bit error rates, etc. Client authentication of sensors to a network is fully automatic using PCA.

**What are the main findings?**

- The problem of numbers of IoT devices and sensors.
- Fully automatic client authentication of sensors.
- Engineless physically unclonable function.

**What are the implications of the main findings?**

- A Root-of-Trust on TLS session at the cheapest cost with easy installation.
- Blockchain of sensors and Blockchain of chips.
- Hardware firewall.

**Abstract:**

Inherently, there exists a significant security hole in sensor networks. The majority of sensors are not high-end Internet of Things (IoT) devices with sufficient computing resources. Connected sensors (physical nodes in real networks) are allocated to logical nodes and managed remotely by a supervisor in a virtual network. Data acquired by sensors are then collected by a data center on which artificial intelligence operates. If an adversary spoofs a logical node (e.g., an account in a transport layer security (TLS) session) of a vulnerable sensor on the network, then it can manipulate data input to artificial intelligence. Artificial intelligence cannot verify the integrity of the data input for learning. It is difficult to stop data poisoning with no countermeasures against session spoofing. To avoid session spoofing, physical and logical nodes must be linked seamlessly. One might think this can be achieved by utilizing Hardware Root-of-Trust (HRoT) based on a Physically Unclonable Function (PUF). However, a PUF is based on an expensive System-on-a-Chip (SoC), which has been specifically designed for high-end devices, like expensive smartphones. Many sensors (low-end and middle-end IoT devices) can hardly be protected with existing PUFs. Since the number of IoT devices with a PUF is insufficient to cover the entirety of IoT devices, an attacker can find a vulnerable IoT device with no PUF to perform session spoofing. This is the problem of numbers. To resolve it, we propose Physical Cyber Authentication (PCA). A Blockchain account (a logical node in a TLS session) is anchored to an integrated circuit (IC) chip inside a sensor, allowing Blockchain to manage sensor networks, which provides necessary data to artificial intelligence, thus forming a Blockchain of sensors.

## 1. Introduction

**Figure 1:**
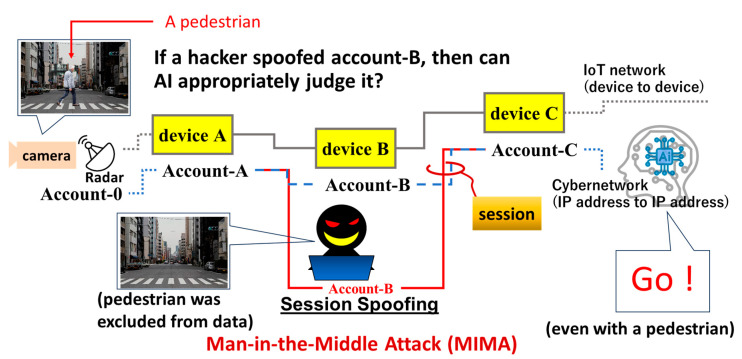
Risk of a Man-In-the Middle Attack (MIMA).

No organization is immune to cyberattacks using session spoofing, no matter how strong its cybersecurity is [1]. Compromised safety of physical networks (i.e., IoT) can result in material damage or even human injuries. There are sensors, such as cameras and radars in autonomous vehicles, that detect and estimate the presence of obstacles on roads. Figure 1 shows sensed data being transmitted through Internet of Things (IoT) devices (A, B, and C) to an artificial intelligence (AI) server. Communications between terminal sensors and device A, devices A and B, as well as devices B and C, and between device C and the AI server are based on transport layer security (TLS) sessions. Devices A to C are allocated to accounts A to C, respectively. The allocation of a device and an account is based on Root-of-Trust (RoT) [2]. There are numerous connected devices in IoT networks, and many of those relay data sensed by IoT sensors (e.g., cameras and radars) to the AI server, such as devices A to C. Some of the devices are out of the control of both the AI server and the autonomous vehicle (e.g., device B). If the security of device B lacks sufficient RoT, then it is vulnerable to an attacker who can easily steal account B for session spoofing. The attacker does not need to break through tough securities and may instead find a vulnerable device among many nodes allocated between the autonomous vehicle and the AI data server. The adversary, having succeeded in session spoofing, can then manipulate communication between the autonomous vehicle and the server. In Figure 1, the attacker removes the pedestrian from the original image. AI then receives data with no pedestrians on the road and instructs the car to move forward. That is how a cyber attacker is able to cause serious injury to a pedestrian by subverting a car. This is an example of the Man-In-the-Middle Attack (MIMA) [3,4].

### 1.1. Session Spoofing and Physical Damage in the Real World

Typical attack targets can be found at any physical nodes with wireless communications, which include radars, cameras, smartphones, construction machines, electric vehicles (EVs), medical sensors for smart healthcare, broadcasting devices, etc. All these IoT devices sense and process data. There are also communication devices that relay data to AI for deep learning. All such devices have a communication module (CM) inside for wireless connections. If an attacker receives a wireless signal from a CM, then an IoT device or the CM itself can become a target. More specifically, Figure 1 depicts an adversary who wants to spoof a session of an account allocated to a target without breaking cryptography. We explain this situation in detail using multiple examples below. To prevent session spoofing, on the other hand, we should protect an account anchored to a target using Blockchain.

**Figure 2:**
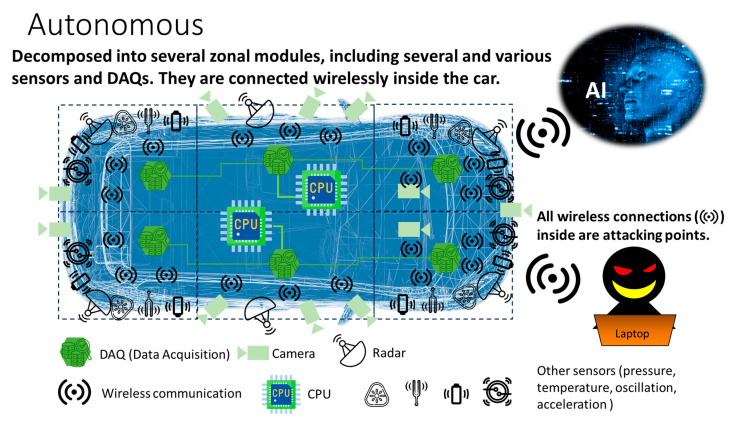
Autonomous electric vehicle (EV), wherein six zones are depicted with dotted squares. In each zone, there are radars, cameras and other sensors and DAQs.

Let us consider a case of autonomous EVs. As illustrated in Figure 2, an electric vehicle (EV) is composed of several zonal modules. In this example, there are six. In each zone, there are several sensors (i.e., IoT sensors) that are connected to and controlled by data acquisition (DAQ), which collects data from sensors and processes it (e.g., truncating noise, etc.) [5,6]. In this example, the six DAQs are connected to two central processing units (CPUs), which further process and transport data to the AI server outside of the EV through a wireless network. The AI server instructs several hundred electronic control units (ECUs) inside the EV on how to drive it. One might think that the targets are in the outgoing wireless EV communications. Hence, we would expect to reinforce the security of devices that relay data out of the EV.

### 1.2. Attacking Points

**Figure 3:**
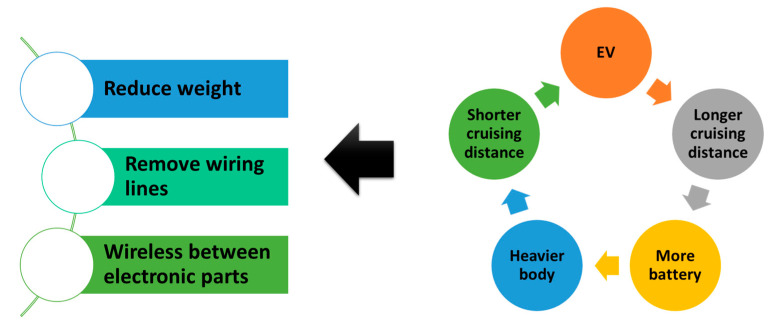
Weight and wireless (EV).

However, there is a wireless network inside the EV as well as the external one. In Figure 3, we illustrate the reason for it. One may want to increase the EV’s cruising distance by installing additional batteries. However, since a battery is a heavy component, the total weight increases with the number of batteries added. This leads to a shorter cruising distance. To compensate for it, an engineer may choose to remove metal wiring lines. This is the reason why wireless IoT networks are installed inside EVs, which are, in turn, composed of many IoT sensors, ECUs, DAQs, CPUs, etc. In other words, there are many cyberattack targets inside an EV. More to the point, devices A to C in Figure 1 are deployed both inside and outside EVs. One might think that wireless networks inside EVs are easier to protect than those on the outside. As the number of connections increases, the frequency of human errors in wiring grows. And there is still no cost-effective solution for RoT covering all devices inside EVs. This raises serious concerns about cybersecurity.

For aviation, reducing the body weight of an aircraft can diminish emissions of greenhouse gas. Replacing wired connections of electro-mechanical controls and entertainment devices inside the aircraft with wireless ones should be conducted without leaving communication nodes vulnerable to session spoofing.

**Figure 4:**
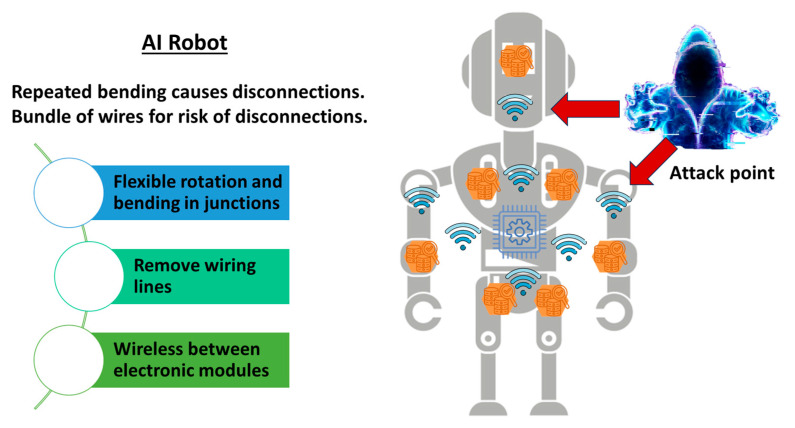
Flexibility and wireless (AI robot).

In the case of robots, as illustrated in Figure 4, there is a different requirement, but it leads to the same demand. Robots may have several joints that bend and rotate smoothly and repeatedly. If there are metallic wires across such joints, then the wires may short circuit after repeated usages. Flexible rotation and bending require removing wiring lines to leave wireless connections across joints, as illustrated in Figure 4. Note that an attacker can identify multiple wireless connections inside robots as potential spoofing targets. We expect that robots with AI—a typical example of physical AI—will compensate for labor shortages. However, the problems regarding attacked targets due to wireless communications inside devices must be resolved first.

Another example exists in medical settings. In operating rooms, there are many wires for electronic equipment, which get in the way of medical staff movements in a small area. Thus, wireless technologies are in demand in medical IoT.

### 1.3. Fake News

**Figure 5:**
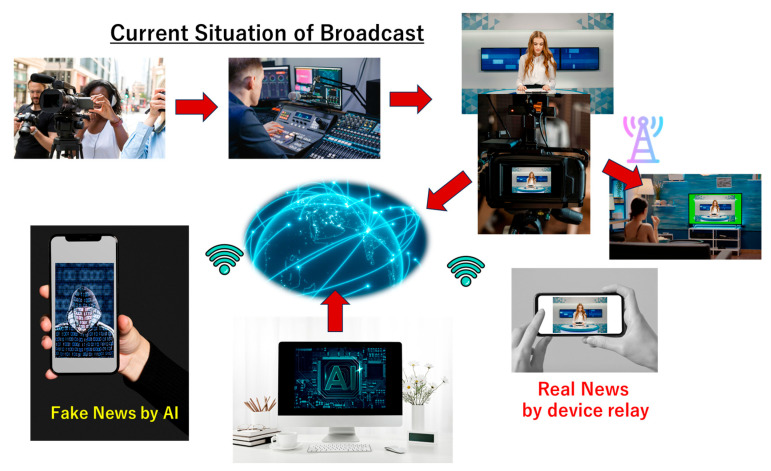
News process and AI’s imitations.

Media suffers from people’s distrust because many cannot distinguish news based on facts from fake news generated by AI. Even though using AI to detect fake news is possible, is there any assurance that said AI is neither compromised nor spoofed? In the example shown in Figure 5, the data of the news source (e.g., video) is collected and recorded by a regular camera. The data is then transferred to and processed by a regular process machine in an editorial room. Then, it can be announced in a studio to be broadcasted or streamed.

People consume news through televisions (TVs), smartphones, etc. Throughout this procedure, staff members spend considerable effort, time and machinery. On the other hand, AI can forge news with or without facts and with minimal effort. Accordingly, the number of AI-generated news stories in the media might increase and eventually overpower news generated in the traditional way. As generative AI’s cost decreases in the near future, the cost of fake news becomes lower than that of regular news. And without countermeasures, the ratio of fake news generated using or by AI will dominate the media entirely.

#### How to Win Back Trust in News

**Figure 6:**
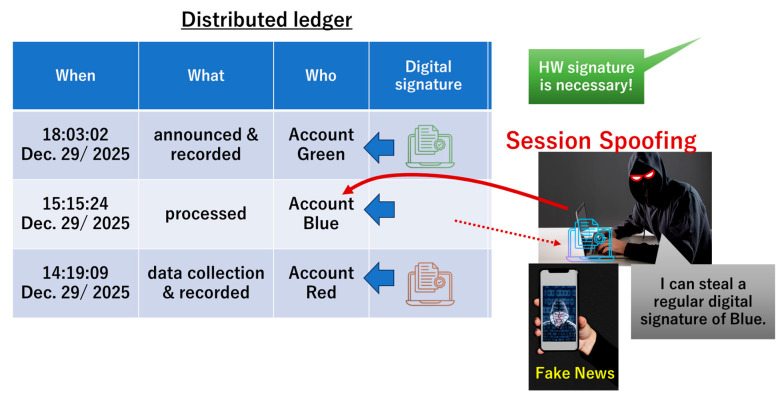
Necessity of a hardware (HW) signature.

This paper does not discuss ethical issues associated with AI-generated news. We focus on technical problems, looking for a way to combat AI imitations. One might think of using a digital signature as part of a solution to a news problem. For example, Figure 6 shows a reporter who collected and recorded data on 14:19:09, 29 December 2025. Her camera recorder is allocated to the red account. The recorded data was transferred to an editorial room and then processed using a regular editing machine allocated to the account blue on 15:15:24, 29 December 2025. Using this data, news was transmitted using regular broadcasting equipment allocated to the green account on 18:03:02, 29 December 2025. The red, blue, and green accounts are allocated to digital signatures red, blue, and green, respectively. The record of the entire process was written and stored in the distributed ledger. One might think that there is no risk of fake news if the distributed ledger is protected by the Blockchain. Note that here, we a priori assumed that a 51% attack is impossible and that cryptography is tough enough (i.e., no quantum computer exists). However, if an attacker successfully spoofs the blue account, then he can use the regular digital signature “blue” and hence manipulate data processing, as illustrated in Figure 1. He can easily stream a fake using a regular media resource. The countermeasures considered in C2PA [7] and CC [8] should be based on a discussion about the session spoofing in Figure 6. The essence is that real devices (A to C) and virtual accounts (A to C) are not tied up seamlessly.

**Figure 7:**
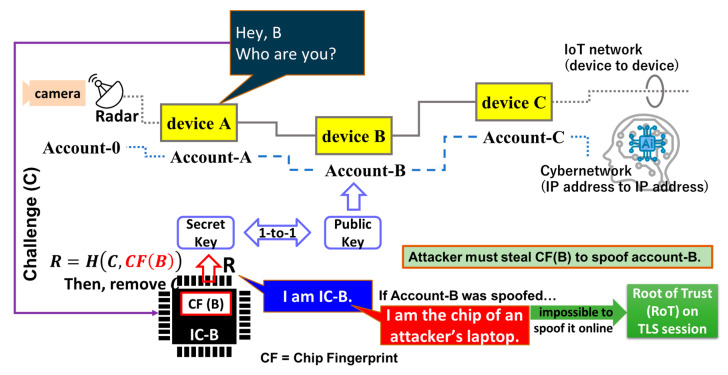
Physical Cyber Authentication (PCA) as a usage model of a chip fingerprint (CF).

As shown in Figure 7, in Public Key Infrastructure (PKI), only a person who has a corresponding secret key can read (decrypt) a message encrypted using a public key. This means that the network address associated with an account can take on the role of the public key. In fact, an address to a Blockchain account is a public key. If the device holds the secret key (e.g., device B in this figure), then account B can be anchored with device B.

IC chips are atomistic components of each device connected to the network (e.g., devices A to C in the figure). If we remove an “atomistic” IC chip from an IoT device, then this device cannot function. If this chip is a frame cache, then the device can no longer process frames.

Suppose that an integrated circuit (IC) chip in device-B has a chip fingerprint, CF (B), and denote this IC chip inside device-B by IC-B. A server or device-A, which wants to identify device-B, asks “Hey B, Who are you?” This is a challenge (*C*) to device-B. Inside device-B, the response (*R*) is generated from *C* and *CF* (*B*) using a hash function (*H*) for the format arrangement of the output response.

(1) R=H(C, CF(B))

**Figure 8:**
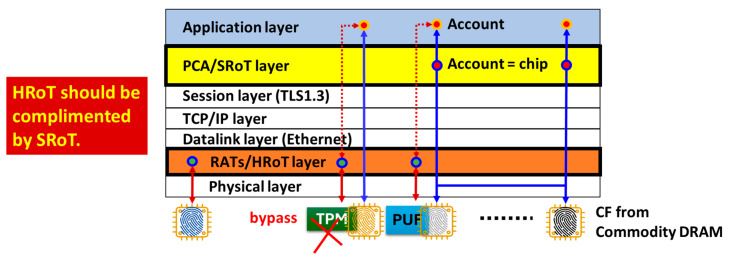
Communication layer with Session Root-of-Trust (SRoT).

This is “I am IC-B” because it has *CF*(*B*) as an argument of the hash function. The secret key and the public key can be generated from this R using an appropriate algorithm. We named this solution Physical Cyber Authentication (PCA) [9]. If device-B was spoofed, then the response would be “I am the chip of an attacker’s laptop”. Therefore, it must be impossible to spoof an account without stealing the device itself. This way, PCA can substantially raise the degree of difficulty in spoofing. Furthermore, since PCA can operate on TLS sessions, we can consider that PCA performs Root-of-Trust on the session, that is, Session Root-of-Trust (SRoT) [9]. Note that the communications in Figure 1 and Figure 7 are performed on TLS sessions. As shown in Figure 8, SRoT exists between the session and the application layers in the communication layer’s hierarchy, while Hardware Root-of-Trust (HRoT) exists between the physical and the datalink layers in this example. This tells us that SRoT can co-exist with HRoT (the existing solution) and hence complement an assurance when HRoT cannot function appropriately for any reason. In (a), the trusted platform module (TPM) [10] is used for HRoT together with SRoT in the same node (i.e., device). In (b), a physically unclonable function (PUF) is used for HRoT together with SRoT in the same node (i.e., a device). The certified device can anchor an account in the application layer by means of HRoT. SRoT anchors the IC chip inside the device to an account in the application layer, as described in Figure 7. Hence, in (a) and (b), this account is protected by two RoTs. Here, SRoT complements HRoT. In (c), only SRoT is used. Most IoT devices or sensors have limited computing resources without PUF or TPM chip installation. Therefore, SRoT can provide such small devices with a practical solution for RoT. SRoT can be applicable to various other usage cases as well.

HRoT is based on the remote attestation procedures (RATS) [11] that run before the OS (of the device to be attested) awakens. The RATS server remotely scans the insides of the device upon booting. This means that the device and the server must establish a connection and exchange data without a security network infrastructure, like a TLS session. This causes vulnerability, as will be discussed in detail in Section 3. With outdated firmware, the TPM can be bypassed, and hence, it cannot satisfy the requirements for HRoT appropriately [2]. This is why SRoT should be implemented to complement HRoT.

**Figure 9:**
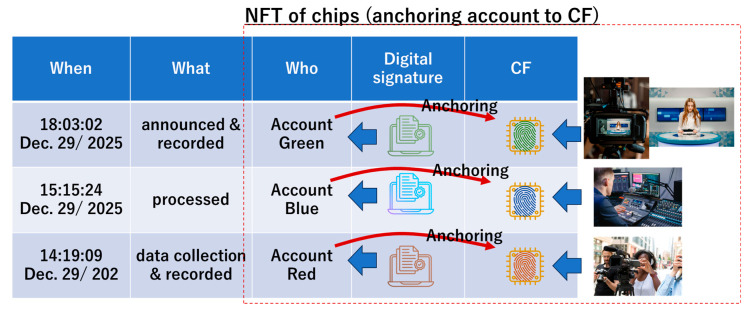
Chip fingerprints and accounts.

Using chip fingerprints (CFs), as shown in Figure 7, we can resolve the problem described in Figure 6. In PCA, the accounts (red, blue, and green) are respectively anchored to the CFs (red, blue, and green) in Figure 9. Since digital signatures are respectively allocated to the accounts, digital signatures and CFs are respectively allocated as well. Without stealing an actual device, an attacker cannot spoof accounts. Digital signatures allocated to accounts finally make sense in the distributed ledger. If the distributed ledger is appropriately protected on the Blockchain, then we can resolve the problem of fake news using PCA. This is similar to having a non-fungible token (NFT) for every chip.

**Figure 10:**
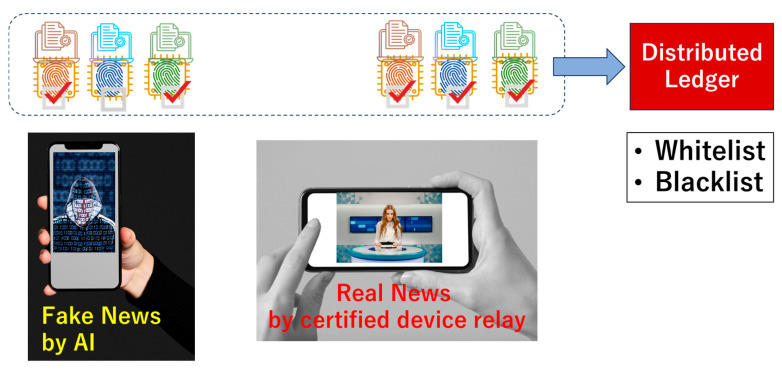
Automatic fake checker, wherein the check marks confirmed that was checked.

In Figure 10, in the news on the right-hand side, all digital signatures (green, blue and red) are checked by the regular CFs (green, blue and red). In the news on the left-hand side, one of the digital signatures (e.g., blue) is not checked by the blue chip fingerprint (CF). Hence, we can judge the news on the left-hand side as not certified, so that the non-certified news and its distributor can be blacklisted. The news on the right-hand side can be regarded as certified and then stored in the whitelist. On the left-hand side, an attacker will likely disguise the blue digital signature anchored with another CF. However, if such a CF is not registered in the distributed ledger, we can judge that the blue digital signature was spoofed and used to deceive the viewer, as illustrated in Figure 5 and Figure 6. By showing if all digital signatures are checked by the regular CFs on display, the automatic fake checker can be proposed.

## 2. True Nature of the Problem and the Way to Resolve It

**Figure 11:**
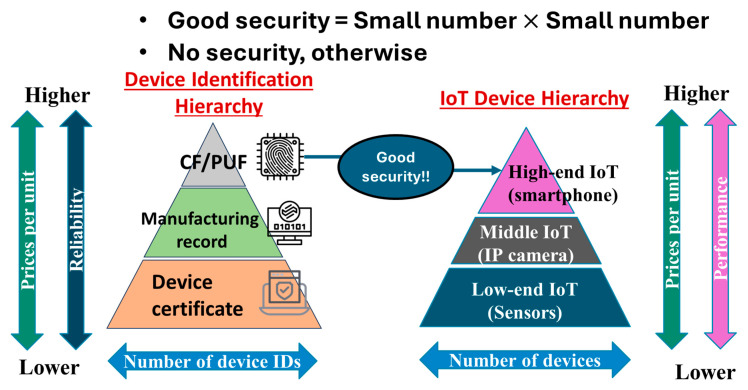
Hierarchies of device identifications and IoT devices. “Good security” signifies the number of devices with sufficient HRoT. “Small number × Small number” is the product of the number of CF/PUF and the number of high-end IoT devices, because the number of CF/PUF is small and the number of high-end IoT devices is small. “No security otherwise” means that IoT devices with no HRoT cannot be protected sufficiently.

Multiple excellent HRoT-based solutions have already been presented to resolve session spoofing. However, it is difficult to distribute them to entire IoT networks. If there are IoT devices with no HRoT in the network, then the network is at risk. An attacker can find a vulnerable IoT device with no HRoT to perform session spoofing. That is, the number of IoT devices with HRoT is insufficient to cover all IoT devices. This is the problem of numbers. Figure 11 shows the device identification hierarchy on the left and the hierarchy of IoT devices (or sensors) on the right. The hierarchy of device identification (left) includes a CF/PUF, manufacturing record, and device certificate, from top to bottom [12]. The device certificate is like a serial number that someone allocates to a device at their convenience. Though the installation is easiest and the cost is lowest, an attacker can easily clone it by analyzing the algorithm to determine the serial number. The manufacturing record is composed of, for example, date of manufacture, location of manufacture (e.g., manufacturing lines, factories, prefectures, nations, etc.), lot number, wafer number, chip number, etc. Indeed, it can be used to identify chips but is not generated from physical randomness. The labeling by manufacturing record is subject to certain rules. Therefore, identification codes generated from manufacturing records can be reproduced if the algorithm is known. On the contrary, if the CF is generated from the manufacturing tolerance of IC chips inside IoT devices or sensors, then we can regard the CF as having been generated from physical randomness because no algorithm can reproduce manufacturing tolerance. The information quantity of physical randomness in CFs increases as the manufacturing of IC chips continues. Such a CF is an ideal seed of IC chip-based PUFs. However, existing PUF solutions are based on specifically designed SoCs that are generally expensive and thus can hardly be applied to middle-end and low-end IoT devices or sensors.

The reliability of device identification is highest and its price per unit are highest at the top (CF/PUF). For cost reasons, most device identifications are based on manufacturing records and device certificates (e.g., serial numbers). The width of the hierarchy, which indicates the number of device identifications, is narrowest at the top and increases going downward because of lower prices and the ease of installation.

**Figure 12:**
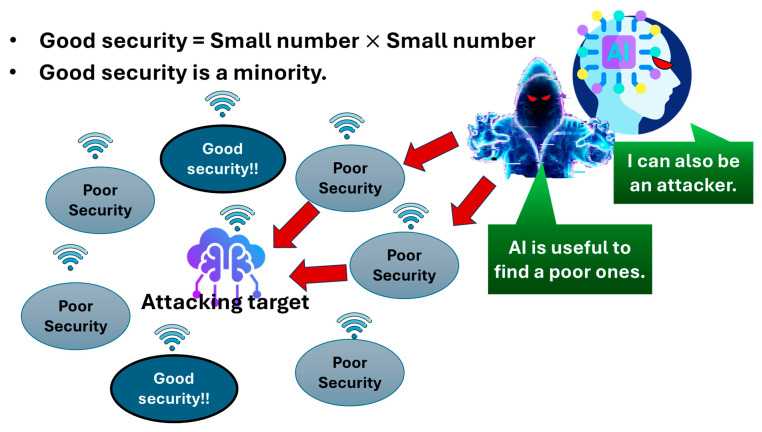
Problem of Numbers in cybersecurity.

In the IoT device hierarchy, the performance and price per unit of IoT devices increases going upward. The width, which indicates the number of IoT devices, increases going downward from high-end to middle-end to low-end IoT devices to balance the cost and performance. In general, high-end IoT devices have a cost leeway to mount an expensive SoC with a PUF inside. Therefore, only high-end IoT devices can ensure good security. The remaining IoT devices or sensors can hardly do so. However, the fraction of stable shipments of high-end IoT devices out of all IoT devices and sensors must be relatively small. That is, high-end IoT devices with good security are in the minority. Therefore, we have the problem of numbers. Figure 12 briefly illustrates the problem of numbers in cybersecurity. An attack target is surrounded by multiple network nodes with good security, as well as those with poor security. As mentioned above, the network nodes composed of IoT devices with good security are outnumbered. An adversary can easily find network nodes composed of IoT devices or sensors with poor security in the vicinity of the target. Moreover, AI may be used by an attacker to identify such a node (low-end or middle-end IoT devices or sensors). Sometimes, AI itself can be an attacker.

### 2.1. Engineless PUF

Cost-effective PUFs, which can be installed in middle-end and low-end IoT devices or sensors with no negative impact on the reliability of the device identifications, are necessary. Without using expensive customized SoCs, we want to utilize the manufacturing tolerance of IC chips, whose shipment number can cover the majority of IoT devices or sensors instead.

**Figure 13:**
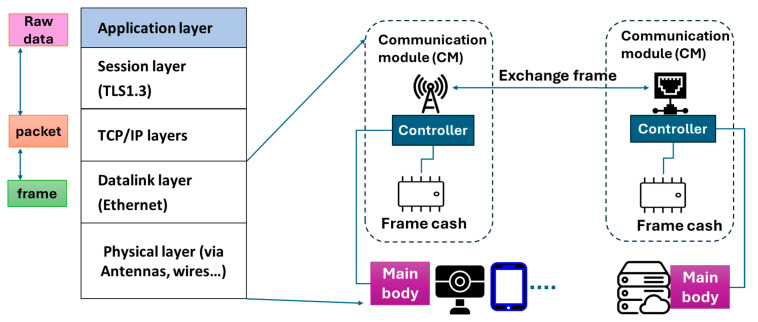
Communications and communication modules.

As previously mentioned, any IoT device or sensor (i.e., connected device) must have a communication module (CM) inside in order to connect to the network. Figure 13 illustrates the operation mechanism of communication modules. Communication modules are typically composed of an antenna, a controller, and a frame cache. In the physical layer, the antenna exchanges frame data with another communication device in another IoT device, sensor or server. The controller then processes the data frame using the frame cache.

Suppose that the communication module receives a frame. The controller then removes an Ethernet header from this frame to make a packet and then forwards it to a processor inside the main body of the IoT device (e.g., IP camera, tablet, sensor, etc.). In the Transmission Control Protocol (TCP)/Internet Protocol (IP) layer, the processor removes the TCP/IP header from the packet. In the session layer, the https header is removed to extract naked data for processing in the application layer. The data processed in the application layer is attached to a different https header in the session layer. It is further attached to a different TCP/IP header for processing in TCP/IP layer. The processed packet is forwarded to the communication module, wherein it is further attached to a different Ethernet header for further processing. In the datalink layer, the antenna sends the processed frame to another communication module in the physical layer.

It is clearly shown that, without the frame cache, IoT devices cannot exchange data on the network. The frame cache is usually a Double Data Rate (DDR)2/DDR3 Dynamic Random Access Memory (DRAM) chip, which is a kind of commodity IC. Since it is not an advanced IC product, it costs, typically, a few US dollars at most. The number of stable shipments of commodity DRAMs must be greater than that of communication modules. Otherwise, some communication modules would be missing a frame cache, and those would not be capable of frame processing. Similarly, the number of stable shipments of communication modules must be greater than that of IoT devices because an IoT device lacking a CM has no means of exchanging data on the network.

**Figure 14:**
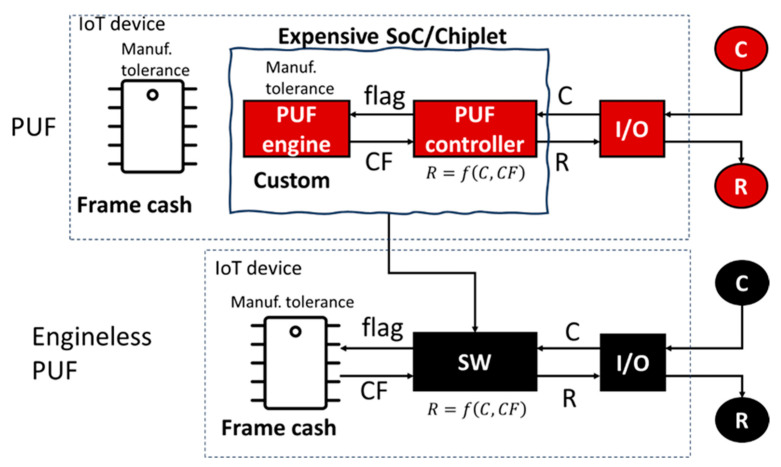
Engineless PUF.

IoT devices with good security are expected to mount a PUF engine, a PUF controller and a frame cache (i.e., DDR2/DDR3 DRAM in the communication module), as illustrated in the first row of Figure 14. Via the external input/output (I/O), the challenge (C) is input to the PUF controller. A flag signal is then input from the PUF controller to the PUF engine to extract the CF using the manufacturing tolerance of the PUF engine. As summarized in Section 2.2, there are several types of PUFs composed of various PUF engines. One is based on circuit delay fluctuations. The others are based on fluctuations in cells, e.g., cell metastability fluctuation or endurance fluctuation. Those fluctuations are attributable to physical randomness in chip manufacturing and hence unrelated to any algorithm. It is preferable that the information quantity of such fluctuations is limitless and stable to environmental change (e.g., temperature change). The CF is forwarded to the PUF controller and then the response (R) is generated from the C and CF using a function therein. The R is then output via the I/O. In some cases, the PUF engine is a custom IC and comprises a chiplet, together with a chip of the PUF controller. In other cases, both the PUF engine and the PUF controller are included in a specifically designed SoC. However, these chips and chiplets increase the cost of IoT device. As illustrated in Figure 11, this causes the problem of numbers (shown in Figure 12).

On the other hand, the frame cache is a commodity DRAM, which also has a manufacturing tolerance. In the second row of Figure 14, we can extract the CF from the frame cache DRAM using its manufacturing tolerance. In this case, software (SW) can replace the PUF engine and the PUF controller so that we can substantially reduce the cost of IoT devices without compromising the reliability of device identifications.

An example of the method to retrieve the CF from a DRAM chip was concretely explained in [13]. A DRAM chip is composed of a memory cell array and peripheral system, which are fabricated on a silicon die of the chip. The memory cell array is further composed of a regular cell array with a smaller area and a redundancy cell array with a greater area. The peripheral system has decoders, controlling circuits, an inner memory composed of fuse or anti-fuse cells, etc. Note that the reliability and longevity of fuse or anti-fuse cells are much better than the cells in memory cell arrays (i.e., DRAM cells). In particular, fuse and anti-fuse cells are very stable during temperature changes.

DRAM chips are mass-produced. In general, some DRAM cells are defective. Those are known as “failure bits”. In quality control, the influence of such failure bits on DRAM chips to be shipped is usually suppressed as much as possible. During the wafer test before the shipment of DRAM chips, the addresses of failure bits found in the memory cell array are stored in the inner memory. After the test, if there is an attempt to access a bit line with a failure bit in the regular cell array, then the periphery system swaps this access to a bit line with no failure bit in the redundancy array. This prevents users of DRAM products from worrying about failure bits.

The peripheral system controls the memory access mode. In the normal access mode, we can only access the regular array. In a special access mode, the redundancy array can be accessed. After the test, first, the normal access mode is used to write all cells in the regular cell array to state 0. Next, the special access mode is used to write all cells in the redundancy cell array to state 1. Finally, we use the normal access mode to read all cells in the regular cell array. Hence, bit lines with failure bits are swapped by bit lines with no failure bits in the redundancy array (state 1) so that we can get a pattern of states 1 and 0. Because failure bits arise in the manufacturing process of DRAM chips, which cannot be controlled, this pattern must be physically random.

Via the external I/O, the challenge (C) is input to the IoT device without a PUF engine. A flag signal is then input from the software to the frame cache DRAM to extract the CF using the manufacturing tolerance of the frame cache DRAM. The CF is forwarded to the software and then the response (R) is generated from the C and CF using a function f therein. The R responds to the outside via the I/O. We call this PCA. As illustrated in the second row of Figure 14, an engineless PUF is a practical solution for PCA. IC chips, from which PCA can extract CFs, are not only limited to DRAM chips but also chips of Pseudo-Static Random Access Memory (PSRAM), Resistive Random Access Memory (RRAM), Magnetic Random Access Memory (MRAM), Static Random Access Memory (SRAM), Ferroelectric Random Access Memory (FRAM), Phase Change Random Access Memory (PCRAM), Flash memories (NAND, NOR, emerging ones) and Main Control Units (MCUs), processors, etc. That is, we can use the manufacturing tolerance of any kind of commodity IC chip. Using PCA, we can achieve the following:

- Securely anchor an account in cyberspace to a commodity IC inside an IoT device, based on the physical randomness in the manufacturing tolerance of the commodity IC.
- Stable shipment of a large quantity of commodity ICs can cover the majority of IoT devices.

### 2.2. PUF Benchmarking

PCA is an engineless PUF that may add a new category to the PUF family because all existing PUFs require a PUF engine [9]. We can compare PCA with existing PUFs. We classify PUFs (incl. PCA) into four groups: (1) SRAM PUF, (2) Circuit PUF, (3) F/AF (fuse/anti-fuse), and (4) PCA.

#### 2.2.1. SRAM PUF

SRAM is composed of memory cells in an IC chip, each of which stores data-0 or data-1 with almost no stand-by power consumption on power-on. However, just on power-on, until SRAM reaches steady-state, the cell array of data-0 or data-1 is an initial value of bits. Such an initial value is physically random due to the manufacturing tolerance of threshold voltages, subthreshold swings, time delays of Field Effect Transistors (FETs) in memory cells, wiring resistances, external voltages and so forth [14,15]. Such physical randomness of initial values is believed to be specific for each IC chip with SRAM.

Since FET performance, characterized by threshold voltages, subthreshold swings, time delays, wiring resistances, etc., is sensitive to environmental changes (like temperature changes) [14,15], an additional countermeasure is necessary to suppress such unwanted sensitivities [16]. There may also be an endurance problem with FETs that compose memory cells. This leads to an additional cost and can hardly make the bit error rate (BER) zero.

The challenge is a chunk of addresses of chosen memory cells. The response is the initial values at those chosen addresses. The information quantity (Info. Q) of the challenge can be determined by the bit number of this chunk and hence linearly increased with that bit number. Therefore, SRAM-PUF is a weak PUF [17]. Direct access from the outside to the SRAM-PUF should therefore be prohibited. There may also a problem of endurance on the FETs that compose SRAM cells.

There are two SRAM subcategories—one is stand-alone SRAM and the other one is SRAM embedded into SoCs. In both cases, it is possible to adopt IC chips with SRAM to act as a PUF engine with a controlled BER.

#### 2.2.2. Circuit PUF

Signal transmissions of two circuits integrated into an IC chip with the same design are generally different in speed due to the manufacturing tolerance of the circuits in IC chips. If we regard a state where one of them has more speed as data-1, and a state where the other has more speed as data-0, then we can extract physical random data from the manufacturing tolerance of IC chips. This is called a delay circuit and is believed to be physically random and specific for each IC chip. Examples of delay circuits are arbiters [18], ring-oscillators [18], glitches [19], etc. FET performance, characterized by threshold voltages, subthreshold swings, delay times, etc., and wiring resistances are, however, sensitive to environmental changes like temperature changes [20]. A couple of measures for it must be appended in IC chips, such as valid maps, majority voting, ECC, etc. [21]. This leads to an additional cost and, nevertheless, cannot reduce the bit error rate (BER) to zero. The PUF engine must therefore be implemented as a custom chip with a controlled BER. There may also be a problem of endurance on FETs that compose delay circuits.

The challenge is an input from switch boxes, which increases by $2^{N},$ with N being the number of switch boxes per unit area. The information quantity of the challenge exponentially increases with the bit number of the challenge. Therefore, the circuit PUF is a strong PUF [17].

#### 2.2.3. Fuse/Anti-Fuse (F/AF)

A PUF composed of an array of fuse (F) cells or anti-fuse (AF) cells can exclude temperature dependence because of the absence of FETs and wiring resistances to generate an output from the PUF. Another advantage is that the longevity and endurance of F/AF cells are very good. This is simply an idea of a random number written to a one-time programmable (OTP) memory. For F/AF to serve as a PUF, we must define how to generate such a random number using dielectric breakdown (AF), electromigration (F), etc.

Each cell is composed of two fuses or two anti-fuses, which are adopted to represent one bit (0 or 1), in a similar way to a circuit PUF [22]. By applying the same voltage on the two fuses on a cell, one fuse breaks earlier than the other (data-1), or the other breaks first (data-0). The array of cells of data-0 or data-1 can be expected to be physically random because the fuse that breaks earlier for each cell is not related to any algorithm under the application of the same voltage. It must be related to the manufacturing tolerance of fuses instead.

Now let us consider an anti-fuse array. By applying the same voltage on the two anti-fuses on a cell, the anti-fuse that breaks earlier than the other is considered data-0, and the other that breaks earlier is considered data-1. The first breakdown of one anti-fuse can reduce the applied voltage to suppress the second breakdown of the other anti-fuse. The array of cells of data-0 or data-1 can then be expected to be physically random because the anti-fuse that breaks earlier for each cell is not related to any algorithm under the application of the same voltage. It must be related to the manufacturing tolerance of anti-fuses instead. An example of an anti-fuse is a dielectric film sandwiched by two metal electrodes. It is preferable to use a hard breakdown of the dielectric film to make the BER zero.

The challenge is a chunk of addresses of chosen cells. In a similar way to SRAM-PUF, we know that the information quantity of the challenge linearly increases with the bit number of the challenge. Therefore, F/AF PUF is a weak PUF [17]. However, if the number of cells is limited, then the ratio of data-0 to data-1 should be about 50% to maximize the entropy of physical randomness. If such a solution is embedded into the SoC, the product yield of the SoC will be determined by the module of F/AF PUF. This means that the inclusion of such PUFs will burden SoC manufacturing. Therefore, it is preferable that the PUF engine of this category is an independent custom IC chip.

Another example of this category is the usage of via holes, in which the depth is targeted to be the same as the designed vertical distance between two electrodes [23].

#### 2.2.4. PCA

The chip solutions of existing PUFs, as mentioned above—SRAM PUF, Circuit PUF, F/AF PUF and so forth—are all PUF engines, as shown in the upper line of Figure 14, from which we can retrieve a physical random code generated from the manufacturing tolerance of chips of PUF engines. When the I/O receives a challenge (C) from the outside, the PUF controller inputs a flag into the PUF engine, retrieves the CF and then generates the response (R) from the C and CF using a function. It is preferable that this function is a hash function.

On the other hand, PCA is an engineless PUF that uses a commodity IC mounted onto an IoT device. The software then takes the role of the PUF controller. When the I/O receives a C from the outside, the software inputs a flag to the commodity IC, e.g., a frame cache in the communication module of the IoT device, and then we can retrieve a physical random code generated from the manufacturing tolerance of the commodity IC chip. The software generates an R from the C and CF using a function (e.g., a hash function). If such a commodity IC is a memory IC, then we can retrieve a physical random code from that F/AF memory that has already been embedded in the peripherals of the memory IC. The embedded F/AF memory is used to relieve failure bits on the memory cell array of the memory IC. Therefore, PCA is excellently stable in the face of temperature changes and is characterized by great longevity.

We performed two experiments to check the temperature stability and longevity of PCA [24]. For the longevity test, we prepared 1116 sample chips of DDR3/DRAM. First, we measured all chips to retrieve 1116 CFs. It was found all CFs were different. Next, we baked all chips at 125 centigrade for 168 h and then measured all chips to retrieve CFs again. This test condition is consistent with a 10-year longevity test of flash memory. It was found all CFs remained the same with no bit shifts before and after the bake. This also shows that the BER was zero in the 1116 sample chips.

For the temperature stability test, we prepared 124 sample chips of DDR3/DRAM. First, we chose one of the chips to retrieve its CF at 27 centigrade (i.e., CF1). Second, we measured its CF at 105 centigrade (i.e., CF2). Third, we measured its CFs at −40 centigrade (i.e., CF3). Then, we checked if any bits in CF1, CF2, and CF3 were different. This procedure was repeated to measure CF1, CF2, and CF3 for all chips. It was found that all sample chips showed no change in any bits for a temperature change ranging from −40 centigrade to 105 centigrade. This also shows that the BER was zero in the 124 sample chips.

There are various relief methods, but a common property among them is to store the addresses of failure bits, which was found during the test before shipment and the registration of IC products. The number of failure bits, as well as the addresses, is determined by manufacturing tolerance. Thus, it is physically random [25]. While the bit capacity of memory IC is large enough, it is unnecessary for the ratio of 0/1 of F/AF cells to be 50%. The bit capacity of a commodity DRAM can reach 1 Gbit, which is large enough. In [13], we evaluated the collision probability that two chips output the same response to the same challenge among 100 trillion chips, while there are only 10 failure bits in the memory cell areas of 4 Mb–16 Gb DRAM chips. The information quantity of the challenge can be determined by the bit number of the challenge and hence exponentially increases with that bit number. PCA is therefore a strong PUF [17].

### 2.3. Risk of Temperature Instability

The parameters to characterize PUF performances are the feasibility to the number of stable shipments (NSS feasibility), the cost per unit, temperature stability, scalability of information quantity (strong or weak), and longevity. The cost per unit, longevity and scalability of information quantity may be self-evident. NSS feasibility was discussed in the above. Why is temperature stability important to characterize PUF performance?

#### 2.3.1. Certification Error (Reduced Reliability)

It is generally required that a PUF generates the same response in response to the same challenge. Otherwise, we cannot use a PUF for device identification. However, the performance of FETs in IC chips, such as threshold voltage, threshold swing, delay time, etc., changes as the temperature changes. Hence, there is a risk that the PUF gives a different response to the same challenge. This would cause a certification error. That is, even a regular device cannot be certified. To suppress such errors as much as possible, logic and SRAM PUFs must be installed along with various modules, such as valid mapping, majority voting, error corrections, etc. [16,20,21]. This turns out to be an additional cost, even though it cannot perfectly remove certification errors.

#### 2.3.2. Security Risk

If the response change due to a temperature change is predictable, by using ML/DL, an attacker can analyze the pattern in the response change so that they can clone PUF performance. Though it is a high-graded attack, improvement in and popularization of ML/DL may reduce the hurdle.

### 2.4. Endurance

A memory cell is likely to degrade in performance during the repeated cycles of reading, programming and erasing. A logic transistor is also likely to degrade in performance during the repeated cycles of accessing and switching. Even though the cycle number is great, if the performance degradations in the level of dies are within an allowable range, then the endurance of such IC products can be regarded as good, that is, tolerant or endurable. Otherwise, the endurance of such IC products can be regarded as bad. In general, logic ICs and commodity memory ICs are designed to have good enough endurance, such as in mass production. However, F/AF is much better in endurance than any kind of memory cells and logic transistors because it does not use FETs. PCA’s endurance is the same as that of F/AF because PCA is based on F/AF cells in the periphery of memory ICs. Therefore, PCA is much better in endurance than logic and SRAM PUFs.

Finally, in this section, we summarize the pros/cons discussed above in Table 1. PCA can have the same performance as F/AF PUFs in temperature stability, longevity and endurance because PCA uses F/AF memory installed inside a commodity IC. PCA costs the lowest because we can exclude a specially designed SoC or chiplet of PUF engines and PUF controllers. PCA is a strong PUF, even though F/AF is a weak PUF. Only PCA can satisfy NSS feasibility because the PUF engine is not necessary in PCA and the number of stable shipments of commodity ICs is much greater than that of IoT devices.

## 3. Root-of-Trust on Session (SRoT)

We also propose a new type of Root-of-Trust [9], which can function based on a TLS session, as illustrated in Figure 8. After opening the session in a conventional way, i.e., successfully exchanging a secret key (e.g., an AES key) between a server and a client (e.g., Client B), we move on to the pre-application process, which runs before moving to the application process. In the pre-application process, the server will send an encrypted challenge (C_2_) to Client B. The encrypted C_2_ is decrypted using the secret key in Client B.

**Figure 15:**
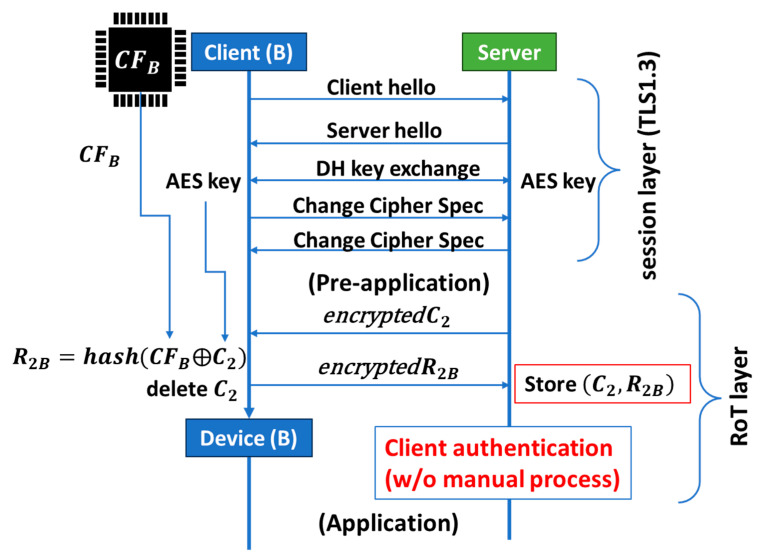
Session Root-of-Trust (SRoT).

Suppose that Client B has PCA so that we can retrieve the chip fingerprint CF_B_ from a frame cache in the communication module of Client B. That is, Client B is an IoT device that can connect to the internet using the communication module. However, Client B is not a high-end IoT device that can additionally install a specifically designed SoC or chiplet of the PUF engine and controller. Client B can retrieve CF_B_ from a frame cache in its communication module and subsequently generate the response R_2B_ from the decrypted C_2_ and CF_B_ using a hash function. The decrypted C_2_ must then be deleted. Client B sends the server the encrypted R_2B_ using the secret key. The server must securely store the set (C_2_, R_2B_) therein. This set is the address of Client B on the network that the server manages. Note that this simple procedure of client authentication has been done automatically. That is, PCA can realize automatic client authentication during a TLS session. This is Session Root-of-Trust (SRoT) [9]. Conventional client authentication is based on certificate authority (CA), such as GlobalSign, VeriSign, GeoTrust, etc. However, they are performed manually, which makes them costly and time-consuming. Inherently, CA was designed to authenticate servers and has been effective because the number of servers is much smaller than that of clients (IoT devices). In IoT networks, however, the number of IoT devices (clients) is much greater, so the need for the automatic process is higher. Since R_2B_ was generated from CF_B,_ which is specific to the frame cache of the communication module of Device B, we can replace Client B with Device B at the bottom of Figure 15. We also successfully performed the pre-application demonstration using DDR4/DRAM mounted on a PC board with Linux Arm. Communications between the sever and device were based on WiFi. We will publish the results elsewhere.

**Figure 16:**
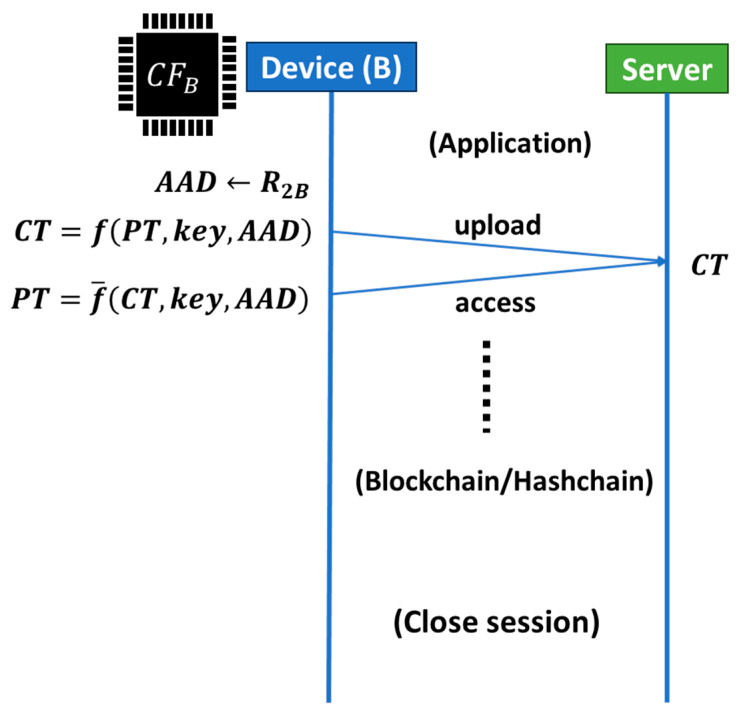
Anchoring of account to device.

Next let us move on to the application process, as illustrated in Figure 16. Client B (Device B) generates a cipher text (CT) from a plain text (PT), the secret key (key), and the additional authentication data (AAD) using an encryption function f and then uploads it to the server. Usually, AAD is an account used in an application. If we use R_2B_ to create an account on the server, we can anchor Device B and the created account on the application layer. Only the account created using R_2B_ can read the uploaded text on the server using the key. In this way, PCA can anchor an account to Device B from which R_2B_ was generated. If we use C’_2_, which is different from C_2_, to generate R’_2B_, then we can anchor a different account to Device B.

**Figure 17:**
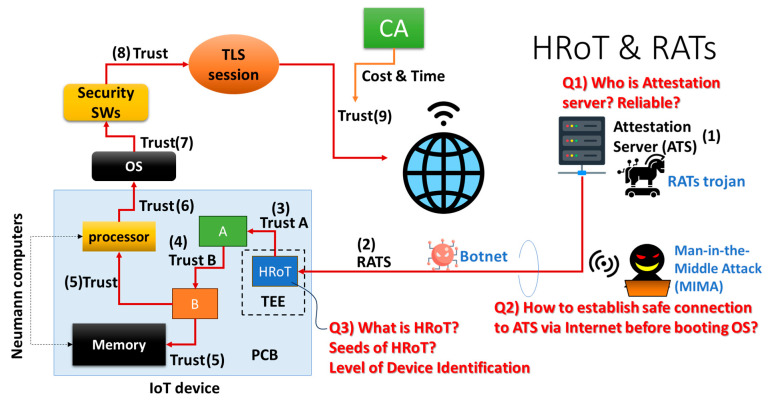
Existing model for HRoT and RATS.

For comparison, we briefly illustrate the conventional Root-of-Trust [2,11] in Figure 17. That is HRoT. There is an IoT device with a PCB board, on which we have a processor, a memory IC (memory), a seed of HRoT (e.g., PUF), and other modules A and B. There is an attestation server (ATS), which will attest to the certification of this IoT device using HRoT on booting the IoT device via the network. However, this network is not based on a TLS session because the IoT device has not booted its OS yet. In other words, (1) an IoT device connects to the ATS before booting the OS at the moment of switch-on so that (2) the ATS can attest the IoT device. This is called remote attestation procedures (RATS). Because there are no cybersecurity tools and a TLS session has not been opened before booting the OS, a specially designed secure environment must be necessary, i.e., the Trusted Execution Environment (TEE), on the system of the IoT device.

Suppose that the RATS succeeded so that we can trust the HRoT inside this IoT device. (3) HRoT can certify and trust Module A, (4) certify and trust Module B, (5) certify and trust the processor with memory (i.e., unit of Neumann computing), (6) certify and trust the OS, (7) certify and trust the security SWs working on the trusted OS, and (8) open a TLS session on which the IoT device can connect an application server having been authenticated by the certificate authorization (CA), such as GlobalSign, VeriSign, GeoTrust, etc.

There are basically three questions.

(1) Who does the attestation server belong to? Is it reliable?
(2) Is there any risk of the spoofing of communication nodes between the attestation server and the IoT device on the RATS? Man-In-the-Middle Attack (MIMA)? Botnet? This is similar to threats related to the session spoofing illustrated in Figure 1.
(3) What is the seed of HRoT? Therefore, a PUF should be adopted for HRoT. However, as mentioned above, there are problems of number and cost. Only high-end IoT devices can adopt a PUF. Even though bypassing is prohibited, the TPM cannot resolve the problem of numbers either.

In addition, there are many steps in RATS/HRoT authentication, as mentioned above in Figure 17. This is the security overhead with energy consumption and extended runtime in booting. Low-end IoT devices (or sensors) may not have sufficient battery volume. An attacker can be granted more time to cryptanalyze a security system during the booting of a device as the runtime becomes longer. Therefore, we prefer to introduce an additional booting step, designed to shorten the runtime in tiny MCUs like DICE [26]. However, DICE is also an additional chip that can be mounted on low-end or middle-end devices. As illustrated on the left of Figure 11, device certification is used to reduce the security overhead in low-end devices. There is a risk that someone maliciously manipulates device certification. This is the reason for Zero Trust [27]. On the other hand, PCA is an engineless PUF and can use a CF based on the physical randomness of manufacturing tolerance of commodity IC chips with no manipulation and with no algorithm. PCA is a solution that is feasible to Zero Trust in low-end and middle-end IoT devices.

A high-end IoT device can also have PCA inside the communication module, as well as PUF/HRoT on the main board, so that we can build both SRoT and HRoT on different layers, as illustrated in Figure 8. The PCA/SRoT layer exists between the application layer and the session layer, while RATS/HRoT (existing solution) exist on the physical layer. SRoT is not an exclusive solution. Hence, those RoTs can compensate for the weak points of each other.

### 3.1. Hardware Firewall

In an application example, PCA is based on a frame cache in the communication module. In another example, PCA can be based on the main memory, which co-works with a processor. In both cases, PCA works after booting the OS, which uses the communication module or the processor. RoT, based on PCA, must therefore work on the TLS session, on which a server can perform automatic client authentication of IoT devices with PCA. High-end IoT devices can have PCA in the communication module and a PUF on the main board. Low-end and middle-end IoT devices can have PCA in the communication module, even though they cannot have a PUF on the main board. This means that only PCA can be widespread and applied to the majority of IoT devices.

**Figure 18:**
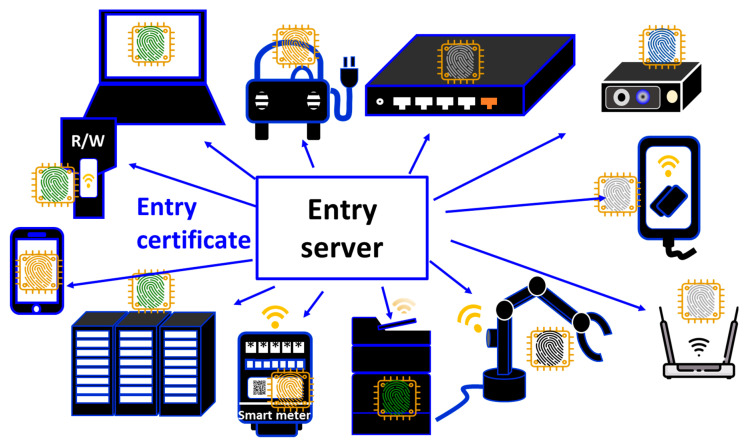
Hardware firewall.

In Figure 18, there is an entry server that handles the entry of all IoT devices under its management. This can be the network composed of various nonhomogeneous IoT devices from the low-end to the high-end by using PCA. This way, we can resolve the problem of numbers, as discussed in Figure 12. Since the entry server can prohibit the entry of IoT devices that have not been authenticated using PCA into this network, the network can act like a firewall. Furthermore, accounts in the firewall can be anchored to IoT devices. That is, the firewall contains the accounts anchored to IoT devices. This can be regarded as a hardware (HW) firewall composed of only authenticated IoT devices [9]. The HW firewall then can clearly define the network boundary composed of only authorized IoT devices, which is indispensable to Zero Trust [27]. As the first step for Zero Trust, we can build a secure enclave on the network using PCA/SRoT.

### 3.2. Application for Post-Quantum Cryptography (PQC)

**Figure 19:**
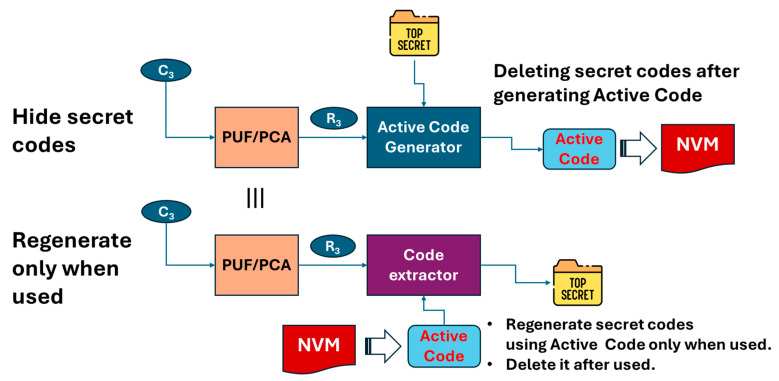
Active code.

Let us discuss an important factor for IoT cyber defense. First, PCA and PUFs can generate a secret code and then regenerate it as necessary using an active code, as illustrated in Figure 19. This includes a PUF or PCA (PUF/PCA), an active code generator, a code extractor and non-volatile memory (NVM).

In “hide secret”, challenge C_3_ is input to PUF/PCA, and then PUF/PCA outputs response R_3_. The active code generator receives R_3_ and the secret code (“Top Secret” in the figure) to generate and output an active code. Then, the secret code must be deleted to avoid theft, while the active code must be stored in NVM.

In “regenerate only when used”, challenge C_3_ is input to PUF/PCA, and then PUF/PCA outputs response R_3_. The code extractor receives R_3_ and the active code from NVM to generate and output the secret code. Then, the secret code is deleted after being used.

“Secret codes”, as mentioned above, are used as secret keys or private keys for symmetric or antisymmetric cryptosystems. Alternatively, they are the codes used to generate or relate to a secret key or private key, etc. This can play a central role in cyber defense systems.

If the secret code was stored in NVM, then an attacker would have a chance to harvest it. Even though the secret code was encrypted, no matter how strong the used cryptography is, future quantum computers are expected to decrypt it. This is a “Harvest (or Steal) Now and Decrypt Later” (HNDL) attack [28]. Therefore, the post-quantum cryptography (PQC) is strongly demanded [29].

A lattice-based cryptography is expected to be a promising solution for PQC [30]. However, we should recall that RSA was considered to be safe, even with quantum computers, before Shor’s algorithm was found [31]. If a new algorithm for quantum computers is discovered, then there is no guarantee that lattice-based cryptography is tough enough to combat that new algorithm. A hash-based cryptography is also considered to be able to withstand Shor’s algorithm and Glover’s algorithm by increasing hash length. However, it cannot be guaranteed that it is tough enough for a new algorithm discovered in the future. This means that it is not a good idea to develop a countermeasure for quantum computers with only software. We believe that it would be effective to hide a secret code from being harvested. PUF/PCA is not based on any algorithm; it is based on physical randomness from the manufacturing tolerance of IC chips. Since we do not need to store secret codes in NVM using PUF/PCA, we can substantially suppress the risk of an HNDL attack. PUF/PCA should collaborate with PQC. However, note that only PCA can resolve the problem of numbers, whereas a PUF cannot, as mentioned above.

### 3.3. Resilience

**Figure 20:**
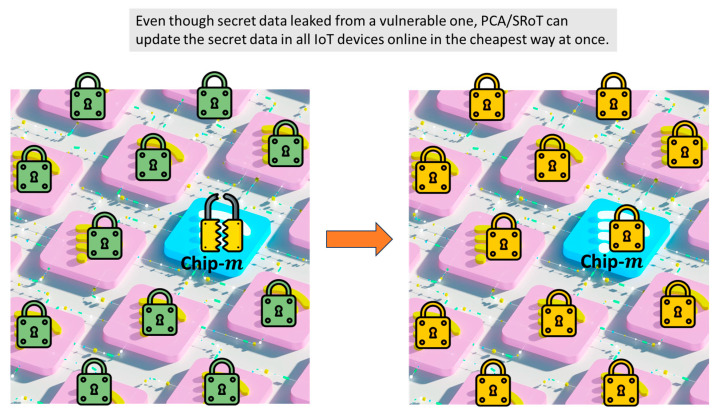
Automatic replacement of secret codes.

Even with a perfect system, there is always the chance of human errors or malfunction of devices in installment. One might copy a secret code of an IoT device and store it in a data traveler, NVM, portable storage, etc. Another might use an IoT device with an imitation of PUF/PCA, wherein, e.g., a pseudo-physical random number is used. Or NVM, wherein an active code is stored, could be broken, and hence there is no way to regenerate a secret code, and so forth. There may be many various cases where we would need to replace a secret code with a new one. PCA/SRoT can perform this automatically, as illustrated in Figure 20.

**Figure 21:**
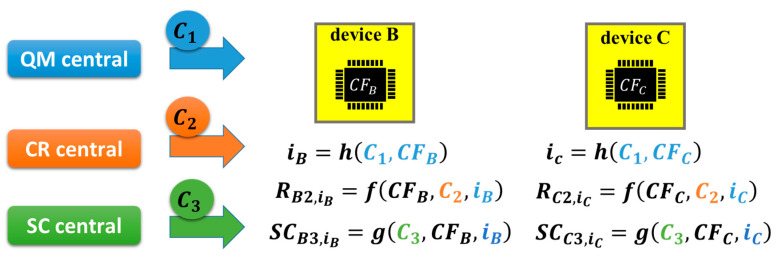
Separation of three digital powers.

**Figure 22:**
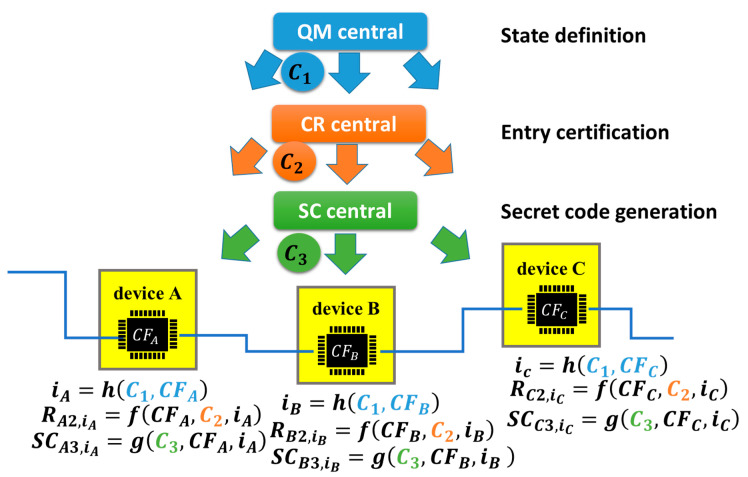
Before code change.

**Figure 23:**
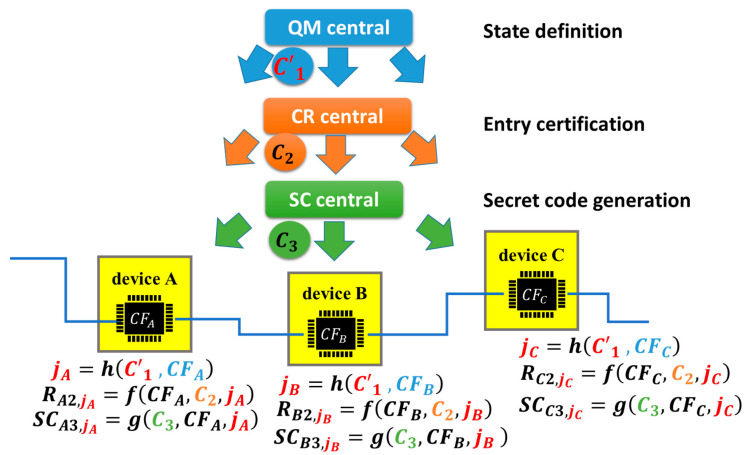
After code change.

On the left of Figure 20, for example, a secret code is leaked from chip-m in IoT device-m. There is a possibility that another secret code was leaked, or will be leaked, from another chip in another IoT device because of the same reason. Therefore, the system supervisor wants to replace all secret codes in all IoT devices as soon as possible. How many IoT devices are in the network under his management? Where are those IoT devices? Even though he can monitor data transactions between accounts or IP addresses, he should know the locations of IoT devices by another method. Note that some IoT devices are mobile. In such a situation, what human resources are necessary to replace all secret codes? How many days and how much is necessary to replace all secret codes? Since a PUF has the problem of numbers, only PCA has the potential to replace all secret codes in all IoT devices no matter how great the number is, as illustrated in Figure 20. The method is described in Figure 21, Figure 22 and Figure 23.

### 3.4. Separation of Digital Three Powers

In Figure 21, the basic mathematical structure is illustrated, wherein there are three central servers—quantum mechanical (QM) central, challenge–response (CR) central and secret code (SC) central. QM central is a central server that managed the states of IoT devices. Note that this does not mean using quantum computers. CR central is a server that manages challenge–response procedures for the entry certification of IoT devices, that is, the entry server in Figure 18. SC central is a central server that remotely manages the generation of secret codes in IoT devices.

An IoT device (device α) receives a challenge C_1_ from QM central and then generates response $R_{\alpha 1}$, where α is B or C in Figure 21 and ${CF}_{\alpha }$ is a chip fingerprint retrieved from chip-α in device α. Chip-α is a commodity IC chip, for example, a commodity DRAM chip for the frame cache in the communication module of device-α, as mentioned above. Subsequently, we can generate a state index $i_{\alpha }$ from $R_{\alpha 1}$ using function *h*. As an example, function *h* may be a hash function. Regarding $R_{\alpha 1}$ as a function having arguments *C*_1_ and ${CF}_{\alpha }$, we have the following equation.

(2) $$i_{\alpha }=h\left(C_{1},\;{CF}_{\alpha }\right).$$

In (4) of [32], the function to define the state using QR central has only one argument, that is, $C_{1}$. On the other hand, we assumed a hash function having two arguments ($C_{1}$ and ${CF}_{\alpha }$) in (2). This means that the states were updated to be different in different devices, while the states are the same in all devices in [32]. By this update, an attacker would be forced to break all the states in all devices, which increases the difficulty level of the cyberattack.

Subsequently, device α receives challenge *C*_2_ from CR central and then generates $R_{\alpha 2,i_{\alpha }}$ for state $i_{\alpha }$ using hash function *f*.

(3) $$R_{\alpha 2,i_{\alpha }}=f\left({CF}_{\alpha },C_{2},i_{\alpha }\right).$$

This is an automatic client authentication, as illustrated in Figure 15. The set $\left(C_{2},R_{\alpha 2,i_{\alpha }}\right)$ is stored in CR central (i.e., the server in Figure 15 and the entry server in Figure 18) to perform the device identification of device α and can be used to manage the address of device α in the HW firewall.

Subsequently, device α receives challenge C_3_ from SC central and then generates $R_{\alpha 3,i_{\alpha }}$ for the state $i_{\alpha }$ using function *f*.

(4) $$R_{\alpha 3,i_{\alpha }}=f\left({CF}_{\alpha },C_{3},i_{\alpha }\right).$$

Then, we can generate ${SC}_{\alpha 3,i_{\alpha }}$ from $R_{\alpha 3,i_{\alpha }}$ using hash function *g* for state $i_{\alpha }$.

(5) $${SC}_{\alpha 3,i_{\alpha }}=g\left({CF}_{\alpha },C_{3},i_{\alpha }\right).$$

This is the generation of a secret code, as mentioned above, which is a secret or private key for a symmetric or antisymmetric cryptosystem, a code to generate or relate to a secret or private key, etc. Such secret keys can also take the central role of cyber defense systems. We can hide such secret codes using the active code and regenerate it only when needed, as illustrated in Figure 19. It is preferable that $C_{2}$ and $C_{3}$ are different because there is a risk that an attacker succeeds in getting $C_{2}$ from CR central, even though stolen $C_{2}$ can be replaced by a new one in a timely manner by using QR central, as illustrated below.

Figure 22 illustrates a part of the network composed of devices A, B, and C, where α=A, B, C in the above equations. Those devices are shown in Figure 1 and Figure 7. QM central generated state-$i_{\alpha }$ in devices A, B and C using C_1_. CR central performed the device identification of devices A, B and C using *C*_2_ for the state-$i_{\alpha }$. In the example shown in Figure 7, device A takes the role of CR central. SC central generates secret code ${SC}_{\alpha 3,i_{\alpha }}$ using *C*_3_ at state-$i_{\alpha }$.

Like the discussion using Figure 20, let us suppose a secret code was leaked from chip-m in IoT device-m. This m is sometimes one of A, B, and C; otherwise, it is neither A, B, nor C. There is a possibility that another secret code was leaked or will be leaked from another chip in another IoT device for the same reason. Therefore, the system supervisor wants to replace all secret codes in all IoT devices as soon as possible. He uses QM central again with a different challenge *C*′_1_ from the previous *C*_1_, that is, a code change, as illustrated in Figure 23.

By performing the same procedure explained using (2) to (5) we obtain the following equations.

(6) $$j_{\alpha }=h\left({C\prime }_{1},{CF}_{\alpha }\right).$$

(7) $$R_{\alpha 2,j_{\alpha }}=f\left({CF}_{\alpha },C_{2},j_{\alpha }\right).$$

(8) $$R_{\alpha 3,j_{\alpha }}=f\left({CF}_{\alpha },C_{3},j_{\alpha }\right).$$

(9) $${SC}_{\alpha 3,j_{\alpha }}=g\left({CF}_{\alpha },C_{3},j_{\alpha }\right).$$

$R_{\alpha \gamma ,j_{\alpha }}$ must always be different from $R_{\alpha ,i_{\alpha }},$ with γ=2 or 3, while *C*_1_ and *C*′_1_ are different because PCA, i.e., an engineless PUF, has the same function as a PUF. In other words, PUF/PCA must generate different responses for different challenges. This property is indispensable when using PUF/PCA for device identification. Since ${SC}_{\alpha 3,j_{\alpha }}$ was derived from $R_{\alpha 3,j_{\alpha }}$, it must be different from ${SC}_{\alpha 3,i_{\alpha }},$ which has been generated from $R_{\alpha 3,i_{\alpha }}$ because of the property of PUF/PCA. By this way, the code change by QM central can surely replace an old secret code with a new one automatically (with no manual processes). There is no limitation on the number of secret codes to be replaced. The period of replacement is very short because all processes are completed remotely by the code change using QR central, CR central, or SC central. Since we can generate symmetric keys, pairs of secret and public keys and any code relating to cryptosystems and cyber defense from ${SC}_{\alpha 3,i_{\alpha }}$, we can renew the whole cryptosystem in the HW firewall remotely in a blink of the eye. It should be noted that those three central servers—QM central, CR central, and SC central—must be independent from each other for security reasons and process only predetermined tasks, as described above. They will not touch the context of data transacted between nodes or IoT devices. However, there is the minimum condition that QM central informs CR central and SC central when the state is changes from $i_{\alpha }$ to $j_{\alpha }$ by the code change from *C*_1_ to *C*′_1_, as shown in Figure 23, so that CR central and SC central can renew $R_{\alpha \gamma ,i_{\alpha }}$ and ${SC}_{\alpha 3,i_{\alpha }}$ to $R_{\alpha \gamma ,j_{\alpha }}$ and ${SC}_{\alpha 3,j_{\alpha }}$, respectively. It may be preferable that QM central performs the code change periodically with or without prior notifications. We call this system the Separation of Digital Three Powers (SDTP) [9].

If we construct SDTP now, then the crypto-scheme to protect communications between servers may be based on TLS 1.3. If we construct it after Q-day, then it must be based on some post-quantum cryptographic scheme. This means that SDTP can be available before and after Q-day.

## 4. Compatibility with Distributed Ledger Technology

**Figure 24:**
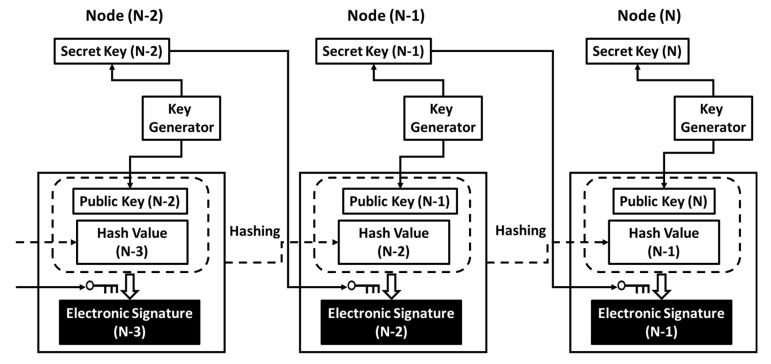
Hash chain without PCA.

**Figure 25:**
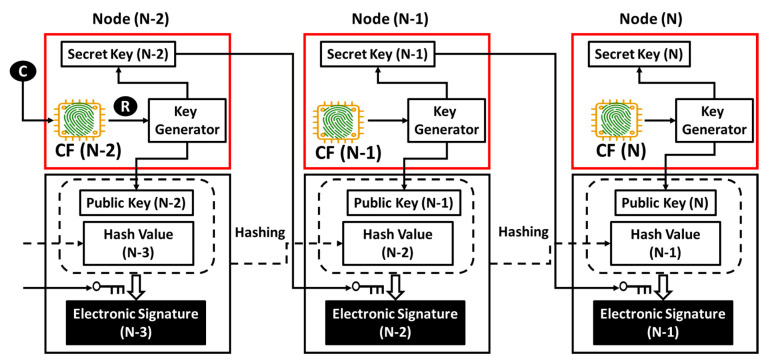
Hash chain with PCA.

An account is a node in cyberspace and logically exists in the network. Data transaction between nodes occurs in a series of hash chains, an example of which is illustrated in Figure 24. A pair of public and secret keys is allocated to each node, and a public key allocated to a node takes the role of the address of the node on the network. The hash value includes a document or some context of digital information to be processed in a node or transferred between nodes (i.e., from a sender to a receiver). In a transaction from Node (N-1) to Node (N), wherein Node (N-1) is a sender and Node (N) is a receiver, Node (N-1) generates Hash Value (N-1) by hashing Public key (N-1) and also Hash Value (N-2) and Electronic Signature (N-2), which have been received from Node (N-2). The sender generates Electronic Signature (N-1) by encrypting Public Key (N) and Hash Value (N-1) using Secret Key (N-1). Then, Hash Value (N-1) and Electronic Signature (N-1) are transferred from the sender to the receiver. The regular receiver can decrypt the electronic signature using the sender’s public key, Public Key (N-1), to check if Node (N-1) really sent Hash Value (N-1) to Node (N). The decrypted electronic signature must include Public Key (N) if Hash Value (N-1) was really sent to Node (N). If the hash chain under study is part of an application on the network, then data transactions between nodes, as illustrated in Figure 24, must occur above the session layer (i.e., TLS session), that is, on the application layer. Figure 7 illustrates an exemplary method in which PCA anchors an account to an IoT device (i.e., Hardware). In Figure 25, we illustrate a method to combine the concepts of the hash chain and PCA illustrated in Figure 7 [25]. Challenge C comes from the outside to an IoT device with a commodity IC (e.g., frame cache DRAM in the communication module or main memory IC on the main board of the IoT device).

According to the procedure illustrated in the bottom line of Figure 14, a software solution for PCA can retrieve the chip fingerprint CF from the commodity IC and then generate and output response R. This R is used to generate a public key and secret key pair, as illustrated in Figure 7. In Figure 21, the secret code is the secret key in Figure 25 or some code to be converted to the secret key. Figure 24 and Figure 25 show networks with and without the commodity IC from which we can retrieve the CF, respectively. Therefore, no modification is required to implement PCA into the formulation of hash chains. That is, PCA is fully compatible with hash chains. If the hash chain under study is part of an application on the network, then data transactions between nodes, as illustrated in Figure 25, must occur on the PCA/SRoT layer that exists between the session layer and the application layer, as depicted in Figure 8.

**Figure 26:**
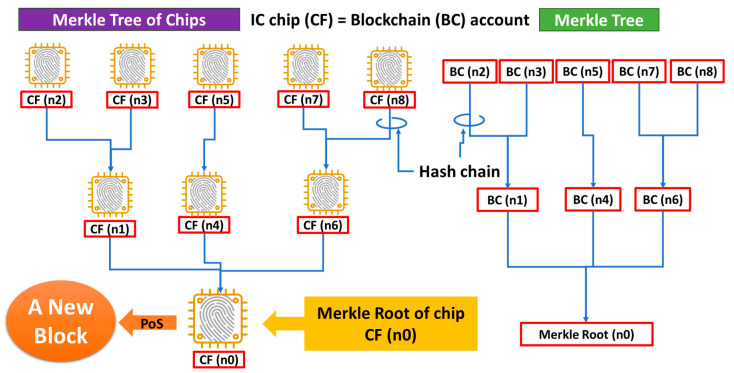
Merkle trees.

In Figure 24 and Figure 25, Node (N-1) received Hash Value (N-2) from Node (N-2). However, another node, e.g., Node (L-2), can also send data, Hash Value (L-2), to Node (N-1). In this event, Node (N-1) can generate Hash Value (N-1) by hashing Hash Value (L-2) and Electronic Signature (L-2), as well as Public Key (N-1), Hash Value (N-2) and Electronic Signature (N-2). This means that data in Node (N-1) come from Node (N-2) and Node (L-2). On the right-hand side of Figure 26, there are three accounts—BC (n1), BC (n2), and BC (n3). Data stored in BC (n1) come from BC (n2) and BC (n3) in this example.

**Figure 27:**
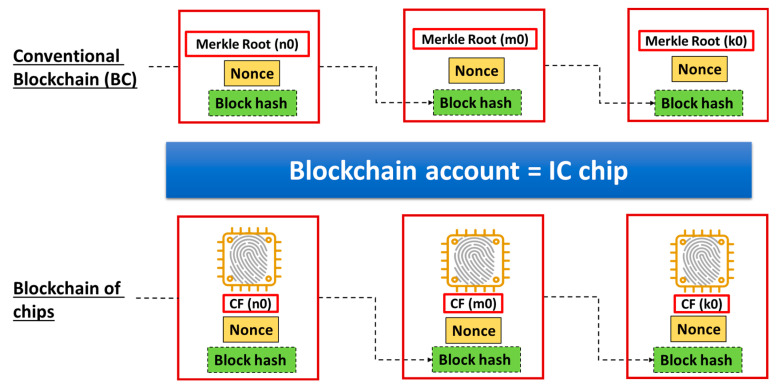
Blockchains.

In a similar way, data stored in an account, BC (n4), come from another account, BC (n5). Data in account BC (n6) come from accounts BC (n7) and BC (n8). Data stored in BC (n1), BC (n4) and BC (n6) are transferred to the bottom of this diagram, that is, an account called a Merkle Root (n0). This way, we can obtain a tree diagram, called a Merkle Tree. By satisfying a condition for Proof-of-Consensus (PoC), the Merkle Root (n0) can be a new block, as shown in the left end of the upper line in Figure 27. After making this block, we can still find another Merkle Root (m0), which can be the next new block, as shown in the center of the upper line in Figure 27. By repeating this procedure, a Blockchain can be grown, as shown in the upper part of Figure 27. With PCA, we can regard account BC (n) as being equivalent to an IC chip with a chip fingerprint CF (n). Therefore, as illustrated on the left-hand side of Figure 26, we have a Merkle Tree of Chips [9] in which BC (n) = CF (n). Therefore, there is a Merkle Root of Chips at the bottom of the tree diagram, with the account being CF (n0). By satisfying a condition for PoC, the Merkle Root of Chips, CF(n0), can be a new Block of Chips, as shown on the left end of the bottom line in Figure 27. After making this block, we can still find another Merkle Root of Chips, CF(m0), which can be the next new block, as shown in the center of the bottom line in Figure 27. Repeating this procedure, the Blockchain of Chips [9] can be grown, as shown on the bottom line in Figure 27. In this example, we used nonce values because of the necessity of checking duplicate payments and monitoring the transaction history. It is self-evident that the applications we discuss in this article are not for cryptocurrency but for IoT cybersecurity. Therefore, PoC should be free from mining to save electricity and time. We should therefore adopt Proof-of-Stake (PoS) for PoC. PCA was, however, designed to be fully compatible with any existing Blockchain. Hence, PCA will not affect any designs or structures of Blockchains.

It is noteworthy to say that we can monitor the history of transactions among IoT devices with PCA and save and share it in a distributed ledger with the protection of the Blockchain of Chips. With PCA, we can regard CF (n) as being equal to Blockchain account BC (n) to build a Blockchain of Chips with no change in the application software for Blockchain. In the IoT network, data transactions occur among memory IC chips. Blockchain is an example application. Therefore, Blockchain works on the application layer in the hierarchy of the communication layer. If a commodity memory IC, which PCA retrieves a CF from, is installed in a communication module or is mounted on the main board of an IoT device, then the OS or some software must have been booted to control the communication module or the memory IC. If the communication module can function with PCA, then the Root-of-Trust via the communication modules can operate. Therefore, we promote PCA/SRoT in this article. On the other hand, RATS must remotely validate a seed of HRoT before the seed completes the validation of the other modules in IoT devices (including a processor). In other words, RATS must be performed without a processor and booting the OS, and hence, HRoT cannot be based on a TLS session. Therefore, as illustrated in Figure 8, the PCA/SRoT layer is independent of the RATS/HRoT layer. PCA/SRoT, which can be installed in communication modules with no additional chips, is not an exclusive solution. This is because a communication module will not prohibit the installation of PUF chips on the main board in high-end IoT devices. Whether or not we can install a PUF chip is the problem of numbers and costs and not the problem of the presence or absence of a communication module. Therefore, PCA/SRoT can coexist with RATS/HRoT by a PUF chip in high-end IoT devices. Note that they reside in different layers in the communication network shown in Figure 8. Nevertheless, there is an option that we can use PCA for the seed of HRoT.

### 4.1. Together with Blockchain—Central Management and Distributed System Powers

After completing the entry certification by CR central at a state managed by QR central, communications between IoT devices can occur securely based on security codes managed by SC central and then generate a record of the communications somewhere, which includes privacy of participants who have regularly entered the HW firewall using certified nodes (or IoT devices). In the conventional model, such a record with participant privacy is saved in and managed by the fourth central server. That is, the fourth central server can know when, how and who exchanged confidential data and manipulated the records even though it cannot read the confidential data.

**Figure 28:**
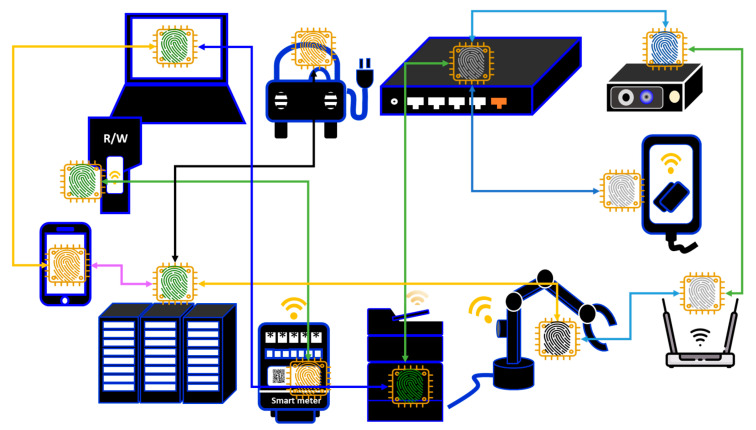
Devices together with Blockchains.

There is a problem of privacy related to saving records of protected communications between certified nodes (or IoT devices) on the fourth central server. The solution for this is the application of Blockchain or related distributed ledger technology, as illustrated in Figure 28. In Blockchain, transaction history is recorded as a hashed digest and then shared by multiple stakeholders. Any central node will not save the record of data transaction history. This is the property of the distributed ledger. The hashing is not decoded; therefore, no one can know when, how and who exchanged confidential data. If an attacker manipulated one of the shared records, the manipulated digest must be different from the others. Therefore, it is easy to detect the manipulation unless an attacker can manipulate more than 50% of all the shared records at the same time. We should build the system (i.e., an HW firewall by PCA/SRoT with Blockchain) to make it hard to manipulate 51% of the shared digests of records simultaneously. Characteristic parameters that reinforce the system are the number of stakeholders, the independence of stakeholders, and the active period of stakeholders. They are the matter of regulations or governance, etc.

### 4.2. Countermeasure for a Ransomware Attack

If an attacker can a enter data system, then he can encrypt valuable data therein so that a regular user cannot use it without paying a ransom. An HW firewall by PCA/SRoT with Blockchain is useful in developing a measure for ransomware attacks because of its many advantages in IoT cyber defense, as follows.

(1) An attacker must steal a device with entry certification to access the data system that he wants to attack, as shown in Figure 7. PCA increases the difficulty of invasion into a secure enclave on the network, i.e., inside the HW firewall, without stealing a certified device.
(2) If a device with PCA, which is necessary to access the data system, was lost, then the supervisor must terminate the entry certificate allocated to the lost device. The attack chance will be terminated with the discovery of device loss.
(3) An attacker wants to manipulate the access record to make it difficult to recover the data system. However, an attacker can hardly hide when (timestamp) and which data he encrypted illegally because it is recorded in the distributed ledger with no manipulation. The accesses using the lost device must be monitored in the unmanipulated record, and then the first timestamp that the lost device accessed (attacking time) will be easily discovered.
(4) The supervisor can easily distinguish processes that other regular users have performed using available devices after the attack from those performed with using the missing device. The supervisor can then backup the data processed using available devices and subsequently recover the system from the previous timestamp.

### 4.3. Social Intelligent System

**Figure 29:**
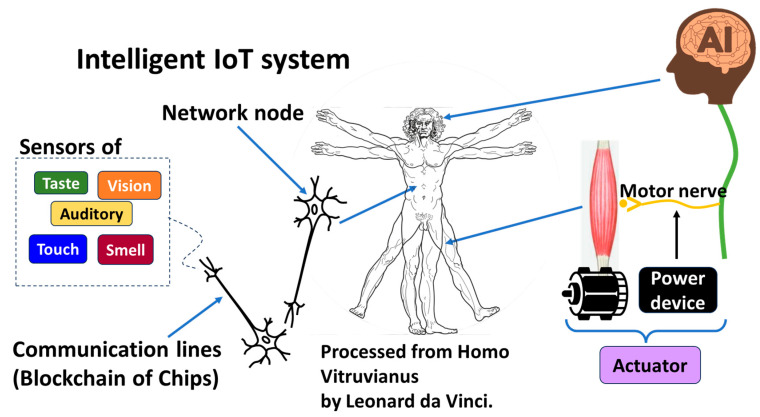
Intelligent IoT.

The human body is a typical intelligent system that intelligent IoT networks imitate, as illustrated in Figure 29. Though current AI is quite different from the human brain, naively, let us regard AI as imitating the human brain in this article. In IoT systems controlled by AI or physical AI systems, power devices then take the role of motor nerves and motors take the role of muscles. Motors and power devices serve as actuators. Network nodes in intelligent IoT systems imitate soma bodies of neurons in the human body. Each node is allocated to an entry certificate by PCA. An axon that transfers a signal from a soma body to a synapsis is a communication line of a hash chain secured by PCA/SRoT in a TLS session. The network of those hash chains with PoC can compose a Blockchain of Chips. Axon terminals can be equipped with IoT sensors, such as taste, vision, auditory, touch, smell and so forth, like the human body. Intelligent IoT systems can collect and provide data to AI automatically. Such a system must be indispensable to any kind of physical AI.

**Figure 30:**
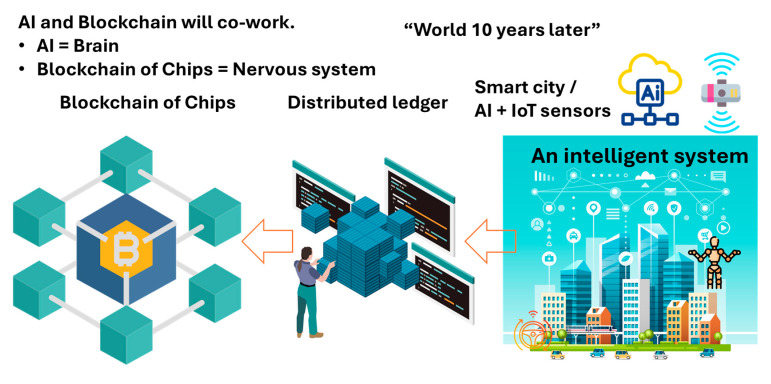
Social intelligent system.

The advanced forms of such intelligent IoT systems may develop for robots, auto-driving public transportation, smart cities, etc., all of which must have s common nature. As an example, Figure 30 illustrates a smart city. It is composed of AI, IoT devices, sensors and relay devices on an IoT network with no exceptions. The IoT network is composed of nodes (IoT devices) and links (communication lines wired or wireless). Since any node can be an attack target and the number of nodes wirelessly connected is huge, automatic client authentication must be indispensable during TLS sessions. Among the relatives of PUFs, only PCA can resolve the problem of numbers. Hashed transaction records of collected and processed data on the network must be saved parallelly in distributed ledgers for privacy reasons and protected by Blockchain to avoid manipulation by adversaries.

As mentioned above, the problem of numbers is the final and most difficult problem in intelligent IoT systems, which conventional PUFs are unable to resolve because low-end and middle-end devices cannot install an additional chip for a PUF engine due to cost constraints. Therefore, PUFs leave poor security nodes vulnerable, as illustrated in Figure 12. PCA is an engineless PUF that can cover the majority of IoT devices and hence create a secure enclave on an IoT network, such as an HW firewall, wherein all nodes can be protected at the same security level of traditional PUFs. PCA/SRoT is the first promising solution in this regard.

The usage models of IoT sensors has been extensively discussed and reviewed in the literature [33,34]. Deployed IoT sensors can collect data for AI; hence, AI can use collected data for active systematic infrastructures [35], essentially behaving like an AI-based active sensor. IoT wearable sensors can be used for elderly care [36], which is expected to make up a significant chunk of healthcare IoT solutions. Furthermore, IoT sensors are likely to be used in smart agriculture development [37,38,39].

## 5. Conclusions

PCA is a new category of the PUF family, namely, an engineless PUF. PCA has the strongest toughness in the family, such as stability in the face of environmental changes (e.g., temperature change), good longevity and endurance, and a zero bit error rate without requiring valid mapping, majority voting, and ECC. Furthermore, only PCA can resolve the problem of numbers because it does not need the additional chip of the PUF engine. Using PCA, we proposed the Root-of-Trust during a TLS session (SRoT), which is independent from and can coexist with the existing HRoTs. We can equate a Blockchain account to an IC chip to create a Blockchain of Chips using PCA/SRoT, such that the data traceability of Blockchain becomes equivalent to chip traceability. This can help improve data integrity, increasing the reliability of data input data into AI. An attacker would be forced to steal a real device to invade a network protected by PCA/SRoT, which increases the difficulty of penetration into a secure enclave on the network. This can also increase the difficulty of record manipulations by gaining access to the data system. Hence, we developed an additional ransomware countermeasure. Since communication lines can be protected by a Blockchain of Chips, we can establish social intelligent systems with safety in mind.

In software engineering, there is no limitation on the number of generated logical accounts. However, we discussed the importance of anchoring a logical account to an IC chip. The shipment number of IC chips is not limitless because ICs are mass-produced. Therefore, the number of logical accounts that can be anchored to IC chips must be limited. The starting point of Zero Trust in IoT systems should be the development of the network of accounts that have been anchored to IoT devices. We emphasize that to consider IoT cyber defense, we should note the problem of numbers.

## Figures and Tables

**Table 1:** Benchmark of PUFs.

PUF	NSS Feasibility	Cost/Unit	Temp. Stability	Strong/Weak	Longevity	Endurance
SRAM	Low	Low	Low	Weak	High	Good
Circuit	Low	Low	Low	Strong	High	Good
F/AF	Low	High	High	Weak	High	Very good
PCA	High	Low	High	Strong	High	Very good

## Data Availability

No new data were created in this study.
